# Interstitial ectopic pregnancy: a case report

**DOI:** 10.11604/pamj.2017.28.135.13889

**Published:** 2017-10-11

**Authors:** Olayemi Atinuke Alagbe, Tinuola Omolade Adeniyi, Olawale Ayobami Abayomi, Emmanuel Olugbenga Onifade

**Affiliations:** 1 Department of Radiology Lautech Teaching Hospital PMB 5000, Osogbo, Osun State Nigeria

**Keywords:** Interstitial, ectopic pregnancy, ultrasonography

## Abstract

Interstitial ectopic pregnancy is a rare type of tubal pregnancy that poses diagnostic challenge. It is associated with the highest risk of massive, uncontrollable bleeding and can result in uterine rupture in the second trimester. This is a rare case of unruptured interstitial ectopic diagnosed in the first trimester by ultrasonography and managed medically with systemic methrotrexate and serial ultrasound monitoring.`

## Introduction

Ectopic pregnancy is the implantation of a fertilized ovum outside the uterine cavity and it's thought to affect 1-2% of pregnancies. 93-97% of ectopic pregnancies are tubal with the interstitial type constituting only 3-4%. This shows interstitial ectopic pregnancy is rare. It is associated with high rate of complications and diagnostic challenge [1]. This is a rare case of unruptured interstitial ectopic pregnancy that was diagnosed by using ultrasonography and managed by systemic methotrexate and serial ultrasound monitoring.

## Patient and observation

**Figure 1:**
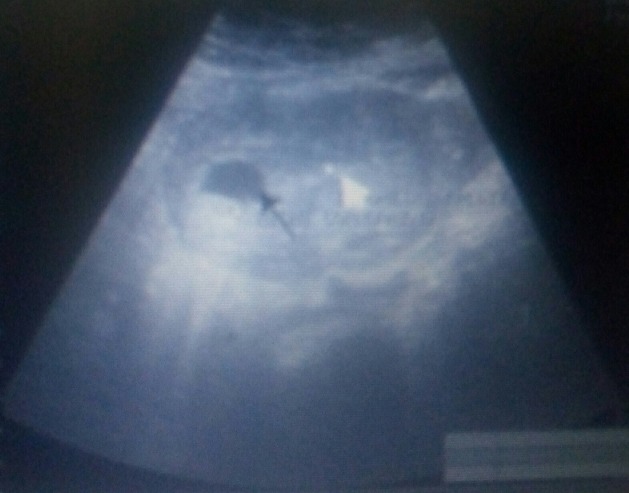
Transabdominal scan of the uterus showing a gestational sac (black arrow) located at the right corneal end separate from the endometrial cavity (black arrow) on transverse view

**Figure 2:**
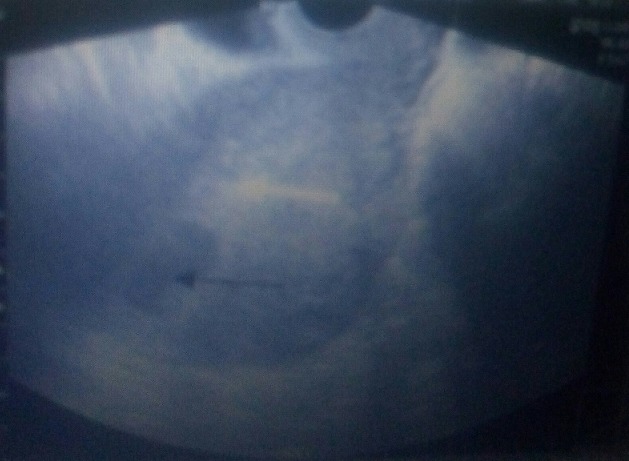
Transvaginal scan of the uterus showing gestational sac (black arrow) located high adjacent to the superior margin of the endometrial plate (white arrow) at the fundus on longitudinal view

A 39 year old gravid 4 para 1 + 2 non alive who was referred to our department for obstetric ultrasound on account of bleeding per vaginum at EGA of 9 weeks 2 days and a provisional diagnosis of threatened abortion She booked at 6 weeks gestation after the pregnancy was confirmed by ultrasound which revealed gestational sac with a diameter equivalent to 7 weeks 1 day and devoid of fetal pole. No associated abdominal pain, trauma, vaginal instrumentation or use of abortifacient. She had two previous spontaneous abortions at EGA of 10 and 8 weeks respectively and was managed by using manual vacuum aspiration. She also had a spontaneous vaginal delivery of a female still birth at 40 weeks with macrosomic baby. She was diagnosed to be diabetic after delivery 2 years ago. She was also diagnosed of multicystic nodular goiter 2 months prior to presentation. She was placed on insulin at booking. Ultrasound at presentation revealed a gestational sac devoid of fetal pole located high at the fundus adjacent to the superior margin of the endometrial plate on longitudinal scan and eccentrically located at the right cornual end on transverse scan (Figure 1, Figure 2). The gestational sac diameter was 2.7cm equivalent to 7 weeks gestation. A diagnosis of right interstitial ectopic was made. Patient was admitted for medical management (using IM methotrexate 75mg stat) and serial ultrasound monitoring. Ultrasound revealed persistent gestation sac on 8^th^ day post methotrexate injection. While, on day 10, the gestational sac has been completely disappeared.

## Discussion

Interstitial ectopic pregnancy is a rare and atypical type of tubal ectopic with a high risk of rupture and haemorrhage compared to other types and it is of increase incidence [2]. It is also a significant cause of maternal morbidity and mortality [3, 4]. It occurs within the interstitial portion of the fallopian tube and therefore has the potential to grow to larger sizes compared to other types of tubal pregnancies by the time of presentation. The interstitial ectopic pregnancy in this case was an incidental finding in the case of a supposed threatened abortion, hence the early presentation. Previous intrauterine instrumentation, pelvic inflammatory disease, previous tubal surgery, previous ectopic pregnancy, assisted reproductive technology and congenital uterine anomalies are risk factors for interstitial pregnancy as is the case for other types of tubal pregnancies [3]. This index case had two previous history of manual vacuum aspiration. Ultrasound features are visualization of a gestational sac or decidual reaction located high in the fundus, with < 5mm of the surrounding myometrium in all planes [5], an echogenic line extending from the mass to the endometrial plate echoe-the interstitial line sign [6], a high sensitivity(80%) and specificity(98%). These signs were seen in the index case. 3D ultrasound is helpful for delineating the gestational sac location. MRI can also reveal the eccentric location of gestational sac to the junctional zone. MRI was not done in this patient due to cost and lack of availability. Interstitial ectopic pregnancy can be complicated by uterine myometrial rupture which usually occur by 2^nd^ trimester and massive hemorrhage. While all ectopic pregnancies are associated with a risk of hemorrhage, interstitial pregnancies are associated with the highest risk of massive, uncontrollable bleeding [7]. There was no rupture or massive hemorrhage in this patient due to early diagnosis. This implies that high suspicion and early diagnosis of interstitial ectopic can forestall complications like massive hemorrhage and uterine rupture. In contrast to the common clinical notion that rupture occurs only between 12 and 16 weeks, in interstitial pregnancies rupture could happen at any time in early pregnancy. Hence, conservative management of interstitial pregnancies should depend on close ultrasonographic follow-up and clinical acumen. Otherwise, rupture could happen suddenly [8]. This patient had ultrasonographic follow up until all the product of conception disappeared completely. Management of interstitial ectopic could be medical with the use of methotrexate, (either systemic or local) or KCL injection or surgical; conservative laparoscopic surgery, uterine artery embolization, cornuectomy or hysterectomy [7]. The latter 2 are usually done in case of rupture or failure of other methods. This patient however responded well to systemic methotrexate.

## Conclusion

Interstitial type is an uncommon type of tubal ectopic pregnancy and delay in diagnosis result in high maternal morbidity and mortality. However, early diagnosis using ultrasonography at early stage of pregnancy prior to rupture and prompt treatment as in this present case can prevent complications like massive hemorrhage and uterine rupture. This case also emphasizes the role of non invasive medical management with serial ultrasound monitoring in the treatment of unruptured ectopic pregnancy.

## Competing interests

The authors declare no competing interests.
